# A Pathophysiological Perspective on the SARS-CoV-2 Coagulopathy

**DOI:** 10.1097/HS9.0000000000000457

**Published:** 2020-08-10

**Authors:** Nuray Kusadasi, Maaike Sikma, Albert Huisman, Jan Westerink, Coen Maas, Roger Schutgens

**Affiliations:** 1 Department of Intensive Care Medicine, University Medical Center Utrecht, Utrecht, The Netherlands; 2 Dutch Poisons Information Center, University Medical Center Utrecht, Utrecht, The Netherlands; 3 Central Diagnostic Laboratory, University Medical Center Utrecht, Utrecht, The Netherlands; 4 Department of Vascular Medicine, University Medical Center Utrecht, Utrecht, The Netherlands; 5 Van Creveldkliniek, Benign Hematology Center, University Medical Center Utrecht, Utrecht, The Netherlands.

## Abstract

Recent evidence is focusing on the presence of a hypercoagulable state with development of both venous and arterial thromboembolic complications in patients infected with SARS-CoV-2. The ongoing activation of coagulation related to the severity of the illness is further characterized by thrombotic microangiopathy and endotheliitis. These microangiopathic changes cannot be classified as classical disseminated intravascular coagulation (DIC). In this short review we describe the interaction between coagulation and inflammation with focus on the possible mechanisms that might be involved in SARS-CoV-2 infection associated coagulopathy in the critically ill.

## Introduction

**Figure 1:**
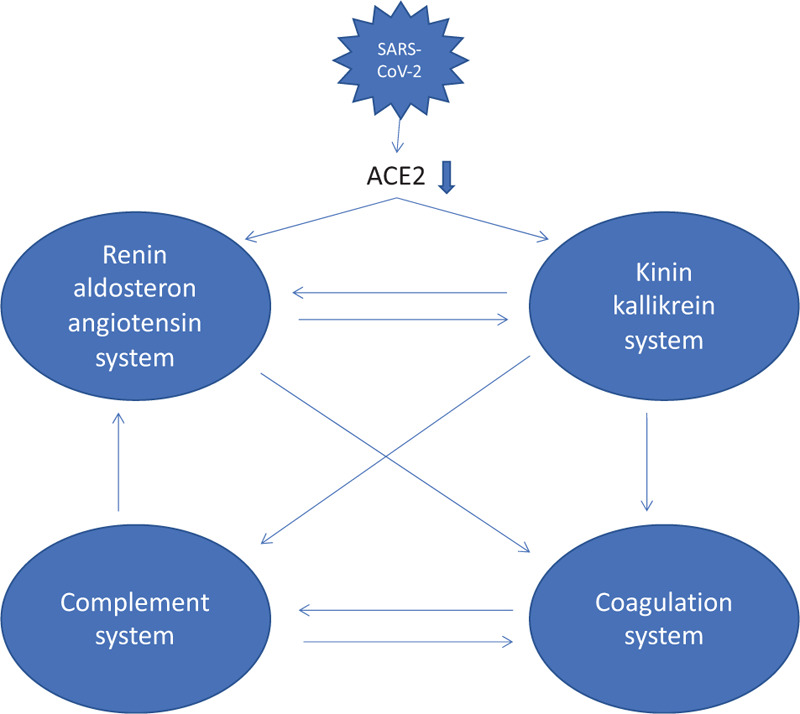
**The 4 interacting pathophysiological mechanisms in SARS-CoV-2 infection**. The potential role of plasma kallikrein in case of SARS-CoV-2 infection may be summarized as (1) the activation of FXII with the end-product thrombin (coagulation system) (2) the generation of bradykinin with subsequent vascular permeability and leakage (kallikrein-kinin system), (3) the activation of the renin-angiotensin system through conversion of renin from pre-renin leading to a pro-inflammatory state through increased angiotensin 1 receptor activation and (4) the activation of C5, in part through activation of C1 via FXII and in part through activation of C3 via plasmin (complement system).^2-6^

Severe acute respiratory syndrome coronavirus 2 (SARS-CoV-2) has crossed the species barriers and is currently causing lethal illness throughout the world. The clinical course in the critically ill infected with SARS-CoV-2 is defined by the development of severe hypoxia based on acute respiratory distress syndrome and multiple organ failure necessitating intensive care support.^1^ From a system biology point of view, at least 4 pathophysiological mechanisms are possibly involved in the pathogenesis of the critical illness associated with SARS-CoV-2 infection (Fig. 1). These comprise (1) the renin-angiotensin, (2) the kallikrein-kinin, (3) the complement and (4) the coagulation system.^2–6^ Angiotensin converting enzyme-2 is considered as the functional receptor for SARS-CoV-2 involved in cell attachment and entry.^7^ The kallikrein-kinin system is currently deemed as a possible route involved in the development of pulmonary edema via the production of bradykinin and upregulation of bradykinin receptors.^6^ Finally, the interaction between nucleocapsid (N) proteins of SARS-CoV-2 and MASP-2, the key serine protease in the lectin pathway of complement activation, resulting in aberrant complement factor 5 (C5) over-activation and deteriorating inflammatory lung injury has been emphasized.^2^

**Figure 2:**
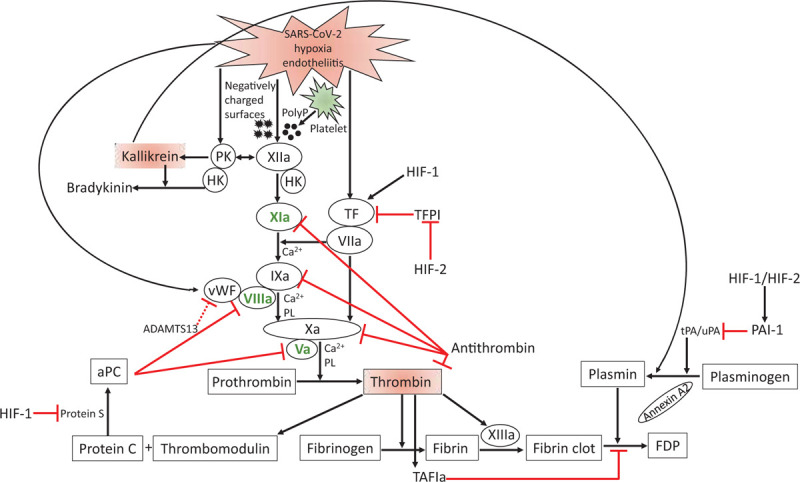
**Schematic overview of the possible mechanisms in SARS-CoV-2 coagulopathy**. Negatively charged particles and activated platelets activate FXII after SARS-CoV-2 infection. PK is converted to kallikrein independent of FXIIa when endothelial cell bounded HK bounds PK. PK generates FXIIa on the surface of the endothelial cells. The binding of FXIIa, PK and HK to endothelial cells results in production of kallikrein. FXIIa activates plasma coagulation pathway via FXIa on the surface of platelets. Together with low ADAMTS13, high vWF and high FVIII levels, the intrinsic tenase complex (FVIIIa:FIXa:FX) leads to increased thrombin generation. Meanwhile, the extrinsic tenase complex (TF:FVIIa), together with the hypoxia induced interactions through HIF-1 and HIF-2 augment thrombin generation. The HIF-1 and HIF-2 stimulate the fibrin clot maintenance by inhibiting PAI-1. Kallikrein not only leads to production of the vasoactive peptide bradykinin, but also stimulates the conversion of plasminogen to plasmin, leading to increased d-dimer production. The feedback amplification of thrombin occurs through FV, FVIII, FXI and platelet activation (green). The natural feedback inhibition by activated protein C (aPC) and antithrombin is given in red. a = activated, ADAMTS13 = a disintegrin and metalloprotease with thrombospondin type 1 motifs 13, Annexin A2 = co-receptor of plasminogen and t-PA, aPC = activated protein C, F = factor, FDP = fibrin degradation product, HIF = hypoxia inducible factor, HK = high molecular weight kininogen, PAI-1 = plasminogen activator inhibitor-1, PK = prekallikrein, PL = phospholipids, PolyP = polyphosphate, TAFI = thrombin activatable fibrinolysis inhibitor, TFPI = tissue factor pathway inhibitor, tPA = tissue plasminogen activator, uPA = urok.

Besides these important mechanisms, recent evidence is focusing on the presence of a hypercoagulable state with development of both venous and arterial thromboembolic events such as pulmonary embolism, deep vein thrombosis and ischemic stroke.^8–11^ The ongoing activation of coagulation related to the severity of the illness in SARS-CoV-2 infected patients is further characterized by thrombotic microangiopathy and endotheliitis as described in autopsies.^12,13^ There are however important reasons to not classify these microangiopathic changes as classical disseminated intravascular coagulation, possibly in part based on local plasma leakage in the lungs.^6,13–15^ Based on evidence from clinical course, SARS-CoV-2 infection associated coagulopathy occurs in two distinct phases. The ongoing coagulation might be caused by prekallikrein dependent contact activation, which is exacerbated by gradually increasing intravascular (micro)angiopathy caused by ongoing endothelial activation. In this short review we describe the interaction between coagulation and inflammation emphasizing the possible mechanisms that might be involved in SARS-CoV-2 infection associated coagulopathy in the critically ill. These mechanisms are summarized in Figure 2 and will be discussed below.

## Normal coagulation

The haemostatic system is based on consecutive activation of precursor proteins.^16^ Tissue factor (TF) plays a pivotal role as initiator of this system in case of vascular injury, known as the extrinsic pathway.^17^ TF expressed after the endothelial cell damage activates coagulation locally.^18^ Coagulation factor (F)VII, present in the blood circulation, contacts with TF forming the extrinsic tenase complex.^19^ This complex initiates thrombin generation but is inhibited by tissue factor pathway inhibitor (TFPI) after sufficient formation of activated FX (FXa). The amplicification loop, known as the intrinsic pathway,^20^ can be activated through feedback amplification of thrombin via FXI, which in turn activates the intrinsic tenase complex (FVIIIa:FIXa:FX) leading to amplification of thrombin generation. The intrinsic pathway can also be triggered directly through contact activation, in which FXII is the initiator. FXII is activated spontaneously when negatively charged surfaces contact the blood^21^ and directly activates FXI. Thrombin in turn stimulates several other factors (FV, FVIII and FXIII) in order to accelerate prothrombin generation and convert fibrinogen to fibrin. Subsequently fibrin clot is formed. Although the role of FXII in bleeding complications seems not to be clinically relevant, its role in inflammation is apparent in acute and chronic inflammation.^22–25^

## Coagulation and inflammation

A systemic infection, like sepsis, is commonly associated with systemic inflammation and activation of coagulation which can result in microthrombosis, a process called immunothrombosis or thromboinflammation.^26–28^ The crosstalk between inflammation and coagulation in sepsis takes place at different levels and is bidirectional with the endothelium being at the center of the process. The interaction between coagulation and activated endothelial cells in sepsis is well defined and is essential for a properly functioning haemostatic system.^29–31^ The extrinsic tenase complex (TF: FVIIa) formed after endothelial cell damage initiates thrombin generation by activating FX or FIX. This tenase complex connects the extrinsic and intrinsic route via FIX. The expression of TF on monocytes, macrophages and endothelial cells is induced by proinflammatory cytokines (Il-1, IL-6 and TNF-α).^32,33^ Increased levels of these proinflammatory cytokines have been observed in severely ill patients infected with SARS-CoV-2.^34,35^ Thrombin results in the recruitment of leucocytes and platelets and stimulates the production of proinflammatory cytokines.^36–41^ Second crosstalk is at the level of the protein C system. Thrombin binds to thrombomodulin on the endothelial cell surface which in turn enhances the generation of activated protein C (aPC).^42^ The aPC acts as anticoagulant agent by inactivating FV and FVIII and has been suggested to act as anti-inflammatory factor by downregulation of proinflammatory cytokine production and decreasing leucocyte adherence and apoptosis.^43^ Decreased levels of aPC results in decreased inactivation of FV and FVIII, which in particular might cause a limited anticoagulant response. The anticoagulant effect of aPC is well defined, however its use in microangiopathic patients, such as in sepsis, did not improve outcome.^44,45^ Third crosstalk is at the level of fibrinolysis where aPC has been suggested to neutralize fibrinolysis inhibitors TAFI and PAI-1 resulting in the activation of the fibrinolysis.^46^ Decreased levels of aPC might in turn limit the fibrinolytic response. The haemostatic system is a complex and dynamic process and continuously subject to changes based on inflammation related factors, especially in critically ill.

## SARS-CoV-2 infection associated coagulopathy

There is plethora of evidence indicating FXII being at the heart of the development of SARS-CoV-2 related coagulopathy in critically ill. It has been shown that negatively charged domain parts are related to the pathogenesis of SARS-CoV-2^47–50^ which in turn might lead to activation of FXII. Prekallikrein (PK) generates FXIIa on the surface of activated endothelial cells, in a receptor-dependent manner. The binding of FXIIa, PK and high molecular weight kininogen to endothelial cells results in generation of kallikrein (Fig. 2). In addition, the release of polyphosphate (polyP) by platelet activation at the site of endotheliitis can be an important driver of coagulation by activating FXII.^51^ In turn, activated FXII initiates the kallikrein-kinin and intrinsic coagulation system resulting in the end-products bradykinin and thrombin, respectively.^52,53^ Therefore, the hypercoagulable state in SARS-CoV-2 might be due to ongoing FXII activation by, amongst others, negatively charged virus particles, activated platelets and the plasma kallikrein-kinin system.

The role of kallikrein in a variety of proteolytic systems, such as the intrinsic pathway of coagulation and fibrinolysis, has sufficiently been emphasized.^54,55^ Since prekallikrein is seen as a potential regulator in the fibrin clot formation through FXII activation and is involved in the pathogenesis of thrombosis, there might be an important role for controlling the hypercoagulable state of the patients infected with SARS-CoV-2 as a therapeutic target.^56,57^ Recently, Shatzel and colleagues emphasized the available evidence, obtained from inflammation models, for the inhibition of the contact activation route as a potential pharmacological target in patients with SARS-CoV-2 infection.^58^

The observed massive coagulopathy in critically ill patients infected with SARS-CoV-2 may also be played by the activation of endothelial cells. The very high plasma levels of FVIII and von Willebrand factor (vWF) and the histological post-mortem analysis of different organs indicate the profound endothelial activation in SARS-CoV-2 associated coagulopathy in critically ill.^9,59^ FVIII and vWF have also been emphasized as being strongly associated with risk of venous thrombosis.^60^ Moreover, a relatively low ADAMTS13 (a disintegrin and metalloprotease with thrombospondin type 1 motifs 13, a plasma metalloprotease cleaving vWF) activity in relation to high vWF antigen may contribute to microangiopathic changes in critically ill patients infected with SARS-CoV-2.^61^ An additional important mechanism contributing to the SARS-CoV-2 associated coagulopathy might be the SARS-CoV-2 infection induced severe hypoxia. Hypoxia has been linked to augmentation of thrombosis.^62–64^ Hypoxia-inducible factor-1 (HIF-1) is activated under hypoxic conditions resulting in upregulation of plasminogen activator inhibitor-1 (PAI-1) and TF as well as inhibition of protein S.^62–64^ Hypoxia inducible factor-2 (HIF-2) is also upregulated and stimulates PAI-1 and inhibits TFPI.^65,66^ Finally, from another angle obesity influences the risk of a hypercoagulable state. Obese subjects have shown to have enhanced coagulation by elevated levels of vWF, TF, FVII, FVIII and fibrinogen, and impaired fibrinolysis by increased secretion of plasminogen activator inhibitor (PAI)-1 and thrombin activatable fibrinolysis inhibitor (TAFI).^67–69^ A significant number of patients infected with SARS-CoV-2 has been described to be obese indicating that being obese may be a predisposing risk factor for the hypercoagulable state. Taken together, all these mechanisms may contribute to the complex hypercoagulable state of the critically ill patients infected with SARS-CoV-2 having severe hypoxia and ongoing endothelial activation.

Surprisingly, the platelet counts, antithrombin (AT), fibrinogen and protein C levels ranged between normal to slightly increased levels in SARS-CoV-2 infected patients who are severely ill.^5,9,70^ These data are in contrast to what is known about evolving disseminated intravascular coagulation (DIC) where platelet counts, AT, protein C and fibrinogen levels are clearly diminished. As the focus of this perspective is restricted to SARS-CoV-2 infection associated coagulopathy in critically ill, the reader is referred to several recent articles emphasizing the differences between DIC and SARS-CoV-2 associated coagulopathy.^71,72^ Moreover, several AT-binding proteins (CD13, CD300f and LRP-1) on human monocytes have been identified and indicated to be involved in blocking the activity of nuclear factor-κβ.^73^ AT also seemed to possess antiviral activity against HIV, HSV and HCV.^74–77^ Therefore, normal to high levels of AT might hypothetically be caused by SARS-CoV-2 infection itself and be due to its possible role in anti-inflammation. Another interesting observation is the increased level of d-dimer during the severe clinical course of SARS-CoV-2 infection.^1,5,9^ D-dimer is a fibrin degradation product. Factors enhancing the degradation of fibrin clot by plasmin might influence the production of d-dimer. Annexin A2, a calcium- and phospholipid-binding protein, is an endothelial surface co-receptor for tissue plasminogen activator (tPA) and plasminogen that stimulates the profibrinolytic state.^78^ There is increasing evidence for a role of annexin A2 at the first phase of viral life cycle known as cell attachment and entry in a variety of viral infections.^79^ SARS-associated cytokines (IL-6 and IFN-gamma) have been shown to upregulate the expression of annexin A2.^80^ The plasma kallikrein is involved in many proteolytic activities. One of the additional functions of kallikrein is to stimulate the production of plasmin.^81^ Plasmin in turn can generate d-dimer from fibrin clots. Although available data is limited, this may imply that factors interfering either with this co-receptor and/or stimulating plasmin might modulate the production of d-dimer.

## Treatment perspectives outside routine anticoagulation

The use of pharmacological thrombosis prophylaxis decreases the risk of thrombotic events in COVID-19.^82^ However, despite the use of thrombosis prophylaxis and therapeutic anticoagulation, the risk of thromboembolic events remained high and appeared not to be sufficient.^8,9^ Pharmacological targeting of the different pathophysiological mechanisms other than routine anticoagulation therapies might improve the severe clinical course seen in patients with SARS-CoV-2 infection at several of them are currently clinically being tested. At the level of platelets, anti-polyP antibodies have the potential to ameliorate the hyperinflammatory and prothrombotic state.^83^ Although successful in animal models,^84^ no human drug currently exists. Blocking FXI or FXII is an appealing option and both have been shown to prevent contact activated thrombosis in animal models. Considering its role in the bradykinin pathway, FXII is the attractive target in SARS-CoV-2 related pathology. From this point of view, blocking FXIIa by its strong inhibitor C1-esterase inhibitor (C1-INH) is of interest and is currently been explored by Osthoff and colleagues at the University Hospital Basel in Switzerland. The potential role of plasma kallikrein in case of SARS-CoV-2 infection may be summarized as (1) the activation of FXII with the end-product thrombin (2) the generation of bradykinin with subsequent vascular permeability and leakage, (3) the activation of the renin-angiotensin system through conversion of renin from pre-renin leading to a pro-inflammatory state through increased angiotensin 1 receptor activation and (4) the activation of C5, in part through activation of C1 via FXII and in part through activation of C3 via plasmin. The inhibition of plasma kallikrein might therefore play a central role as target treatment, especially as 2 drugs are already on the market (lanadelumab and ecallantide). The use of these agents, alone or in combination with other agents acting on complement, coagulation and/or renin-angiotensin systems, might be an attractive treatment strategy. At the level of the complement system, the risk of thrombotic complications in patients with paroxysmal nocturnal hemoglobinuria, a rare life threatening disease manifesting with hemolytic anemia, bone marrow failure and thrombosis, has well been established.^85^ Even more so, treatment with the C5 blocking agent eculizumab decreases these thrombotic risk significantly indicating the inhibition of complement activation as a possible target to reduce the risk of thrombotic complications.^86^ Finally, the interplay between inflammation and thrombosis has already being stressed. Interfering with anti-inflammatory agents, such as tocilizumab, anti-IL-6, anti-IL-1 and others, could have a beneficial effect on the level of coagulopathy as well. Future research to prevent or treat the SARS-CoV-2 infection related coagulopathy should therefore focus on targets other than regular antithrombotic treatment.

## Conclusion

The severe critical illness as seen in patients infected with SARS-CoV-2 is characterized by multiple organ failure and a deep hypoxia related to ARDS and pulmonary embolism. Central in the pathogenesis are the activation of the renin-angiotensin, the kallikrein-kinin, the complement and the coagulation system. Pharmacological interventions aimed at these mechanisms may alter the clinical course in these patients.
